# CD47-retargeted oncolytic adenovirus armed with melanoma differentiation-associated gene-7/interleukin-24 suppresses *in vivo* leukemia cell growth

**DOI:** 10.18632/oncotarget.6292

**Published:** 2015-11-02

**Authors:** Gongchu Li, Hu Wu, Lianzhen Cui, Yajun Gao, Lei Chen, Xin Li, Tianxiang Liang, Xinyan Yang, Jianhong Cheng, Jingjing Luo

**Affiliations:** ^1^ College of life sciences, Zhejiang Sci-Tech University, Hangzhou, Zhejiang, China

**Keywords:** oncolytic adenovirus, CD47, thrombospondin-1, leukemia, interleukin-24

## Abstract

Our previous studies have suggested that harboring a soluble coxsackie-adenovirus receptor-ligand (sCAR-ligand) fusion protein expression cassette in the viral genome may provide a universal method to redirect oncolytic adenoviruses to various membrane receptors on cancer cells resisting to serotype 5 adenovirus infection. We report here a novel oncolytic adenovirus vector redirected to CD47+ leukemia cells though carrying a sCAR-4N1 expression cassette in the viral genome, forming Ad.4N1, in which 4N1 represents the C-terminal CD47-binding domain of thrombospondin-1. The infection and cytotoxicity of Ad.4N1 in leukemia cells were determined to be mediated by the 4N1-CD47 interaction. Ad.4N1 was further engineered to harbor a gene encoding melanoma differentiation-associated gene-7/interleukin-24 (mda-7/IL-24), forming Ad.4N1-IL24, which replicated dramatically faster than Ad.4N1, and elicited significantly enhanced antileukemia effect *in vitro* and in a HL60/Luc xenograft mouse model. Our data suggest that Ad.4N1 could act as a novel oncolytic adenovirus vector for CD47+ leukemia targeting gene transfer, and Ad.4N1 harboring anticancer genes may provide novel antileukemia agents.

## INTRODUCTION

CD47, or integrin associated protein (IAP), is a transmembrane protein which serves as a receptor for thrombospondin (TSP) family members, as well as a ligand for macrophage signal-regulatory protein α (SIRPα) [1]. CD47 has a higher level of expression on acute myeloid leukemia blasts and leukemia stem cells, liver cancer stem cells, as well as pancreatic stem cells, as compared with their normal counterparts [2–4]. The CD47-SIRPα interaction between acute myeloid leukemia cells and macrophages inhibits the phagocytosis by macrophages [5]. Therapeutic antibodies against CD47 selectively eliminated acute myeloid leukemia [6], lymphoblastic leukemia [7], pancreatic cancer stem cells [3], breast cancer [8], and hepatocellular carcinoma [4] through inducing the phagocytosis of cancer cells, directly inducing apoptosis, or sensitizing cancer cells to chemotherapy. In addition, an engineered high affinity SIRPα variant elicited dramatic synergistic effect with various tumor specific monoclonal antibodies through inducing the phagocytosis of cancer cells by macrophages [9]. Furthermore, delivery of anti-CD47 siRNA by nanoparticles inhibited melanoma tumor growth and lung metastasis [10]. CD47 blockade on cancer cells not only induced macrophage phagocytosis, but also activated antitumor CD8+ cytotoxic T cell response [11]. Collectively, CD47 provides a promising target for cancer therapies and have attracted wide interests.

Due to lytic replication, efficient gene transfer, and low pathogenicity, oncolytic adenovirus, or conditionally replicating adenoviruses, has become a promising strategy for cancer therapy [12–15]. In this strategy, adenoviruses are engineered to selectively replicate and induce cytotoxicity in cancer cells. The oncolytic modifications include the deletion of viral genes which are essential to complete the viral lytic cycle in normal cells, but not in tumor cells, or controlling the expression of genes regulating viral replication with tumor-specific promoters [16]. Coxsackie-adenovirus recptor (CAR) is the primary receptor for the infection of serotype 5 (Ad5) adenoviruses, the most commonly used adenoviral vector in cancer gene therapy [17, 18]. However, leukemia cells only express low levels of CAR, which results in resistance to Ad5 infection [19]. To redirect Ad5 to leukemia cells, adenoviruses were modified through genetically incorporating receptor-specific ligand peptides into the viral fibers, or altering viral tropism through the replacement of the fiber knob alone or together with the shaft domain to form chimeric fibers [20–23]. Recombinant adaptor proteins containing CAR and specific ligands were also utilized for bridging Ad5 to various cell membrane receptors [24, 25].

Previously, we designed a novel strategy to redirect oncolytic adenoviruses to leukemia cell membrane receptors though carrying a sCAR-ligand expression cassette in the viral genome [26]. For example, to retarget oncolytic adenoviruses to interleukin-3 receptor α subunit (CD123), a sCAR-IL3 expression cassette was genetically inserted into the viral genome. During viral packaging, the sCAR-IL3 fusion protein would be expressed in packaging cells and noncovalently installed on viral surface, which bridged oncolytic adenoviruses to CD123+ leukemia cells. After infection and replication in leukemia cells, the sCAR-IL3 expression would help newly produced oncolytic adenoviruses to be further modified and infect more leukemia cells. Therefore, harboring sCAR-ligand expression cassette in the viral genome may become a universal method to redirect oncolytic adenoviruses to various membrane receptors on cancer cells resisting to Ad5 adenovirus infection.

In addition to therapeutic antibodies described above, oncolytic adenoviruses may provide an alternative therapeutic method for targeting CD47+ leukemia. In the work presented, we constructed a novel CD47 targeting oncolytic adenovirus through genetically modifying Ad.sp-E1A, a previously reported conditionally replicative oncolytic adenovirus in which the viral E1A is controlled by a survivin promoter [27], to carry a sCAR-4N1 expression cassette in the viral genome, forming Ad.4N1. Peptide 4N1 is the C-terminal CD47/IAP-binding domain of TSP-1 with the amino acid sequence RFYVVMWK [28]. Moreover, Ad.4N1 was further armed with a gene encoding melanoma differentiation-associated gene-7/interleukin-24 (mda-7/IL-24), a well known anticancer agent described previously [27, 29, 30], to form oncolytic adenovirus Ad.4N1-IL24. The *in vitro* and *in vivo* therapeutic effects of Ad.4N1 and Ad.4N1-IL24 against CD47+ leukemia cells were evaluated.

## RESULTS

### *In vitro* characterization of sCAR-4N1 fusion protein

**Figure 1:**
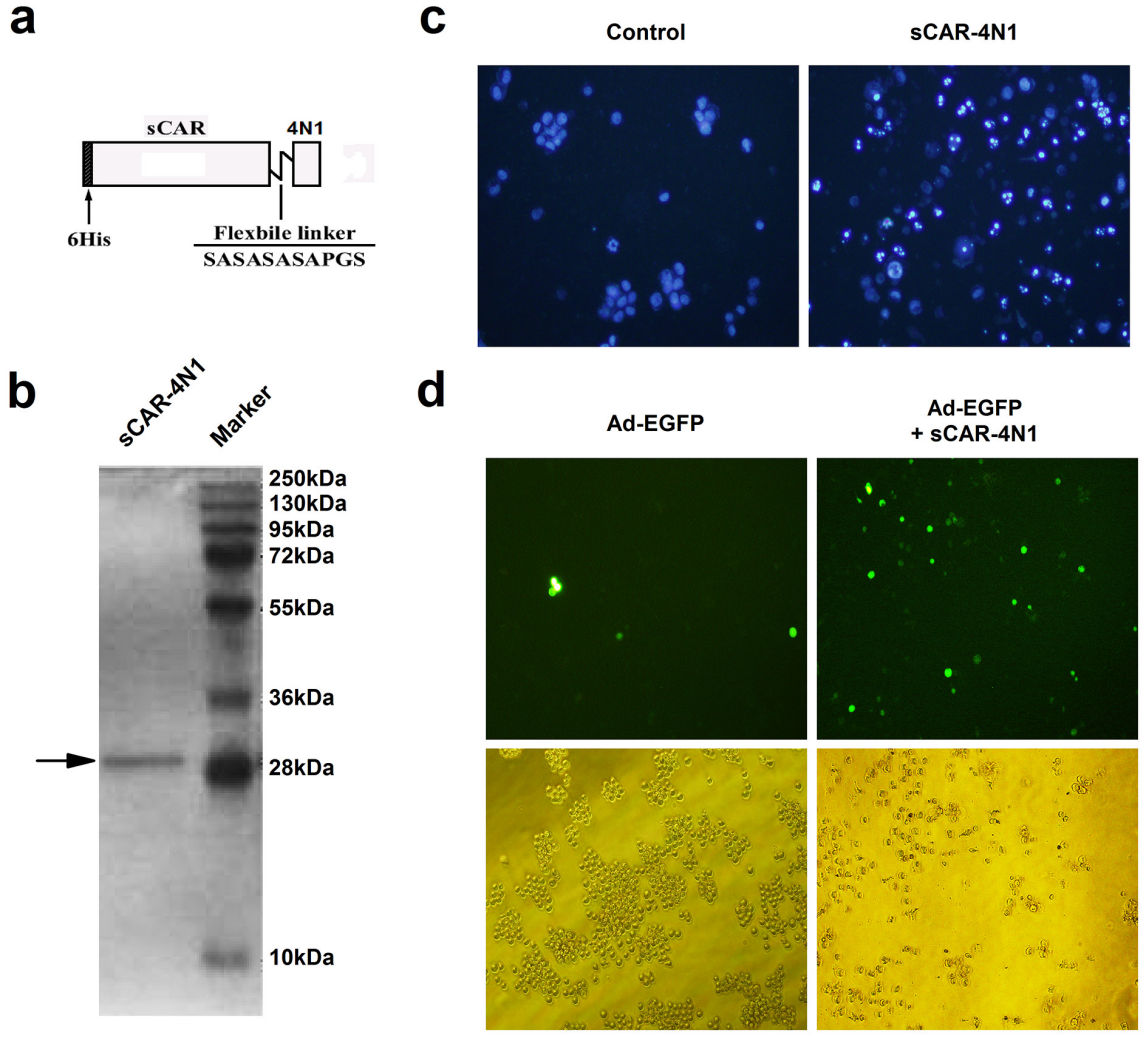
The *in vitro* characterization of sCAR-4N1 fusion protein **a.** Schematic structure of the sCAR-4N1 fusion proteins. The recombinant protein consists of a 6his-tag, an extracellular domain of CAR with 239 amino acids, a flexible linker (SASASASAPGS), and the 4N1 peptide. **b.** The bacterial production of recombinant sCAR-4N1. The sCAR-4N1 was purified through a Ni-NTA-Sepharose column, and subjected to SDS-PAGE followed by Coomassie Brilliant Blue staining. **c.** The cytotoxicity of recombinant sCAR-4N1 to K562 cells. K562 was treated with 20 μg/ml of sCAR-4N1 followed by Hoechst 33342 staining. PBS was used as the control. Nuclear staining and apoptosis were observed under a fluorescent microscope. Shown is a representative experiment from 3 separate repeats. **d.** K562 cells at 2.5 × 10^5^ cells per well were treated with 4 × 10^8^ viral particles (vp) of Ad-EGFP pre-mixed with 10 μg of sCAR-4N1. Cells treated with Ad-EGFP alone served as the control. After 2 days, GFP-positive cells were analyzed under a fluorescence microscope. Shown is a representative experiment from 3 separate repeats.

Recombinant sCAR-4N1 protein was designed to contain a 6his-tag, a human coxsackie-adenovirus receptor extracellular domain (sCAR), a short flexible linker, and a TSP-1 C-terminal 4N1 peptide (Figure 1A). The expression and purification of sCAR-4N1 from a bacterial expression system were examined by SDS-PAGE followed by Coomassie Brilliant Blue staining. As shown in Figure 1B, a relatively pure protein with expected molecular weight was obtained. To test the activity of sCAR-4N1 fusion proteins, CD47+ leukemia cell line K562 was treated with sCAR-4N1 followed by Hoechst 33342 staining. PBS was used as the control. As compared to the control, sCAR-4N1 treatment dramatically induced apoptosis in K562 cells (Figure 1C). Furthermore, K562 cells were treated with Ad-EGFP, a replication-defective adenovirus expressing enhanced green fluorescent protein, combined with sCAR-4N1. K562 cells treated with Ad-EGFP alone served as the control. As determined by fluorescent microscopy (Figure 1D), sCAR-4N1 significantly increased the Ad-EGFP infection in K562 cells. Therefore, our results determined that sCAR-4N1 fusion protein could not only induce apoptosis, but also facilitate adenoviral infection in K562 cells.

### Oncolytic adenoviruse carrying sCAR-4N1 expression cassette elicited cytotoxicity to CD47+ leukemia cells

**Figure 2:**
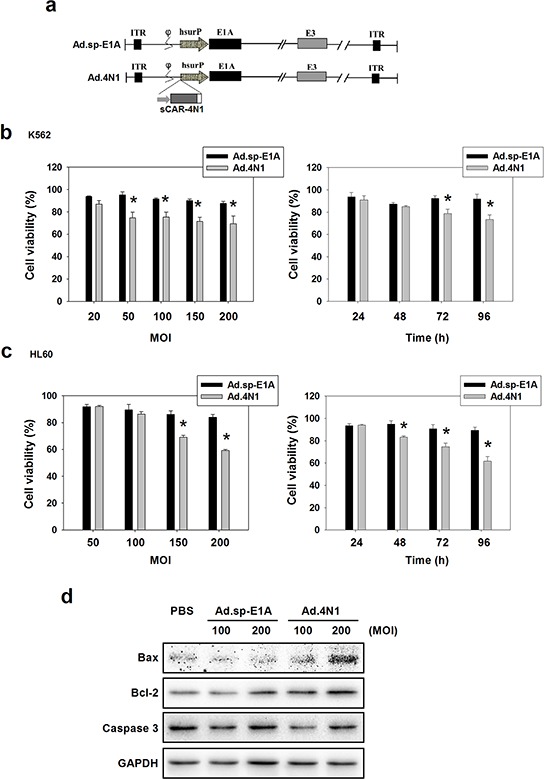
*In vitro* characterization of oncolytic adenovirus Ad.4N1 **a.** Schematic structure of Ad.sp-E1A and Ad.4N1. As shown in Ad.sp-E1A and Ad.4N1, the viral E1A promoter was replaced by a human survivin promoter (hsurP). Ad.4N1 contains a sCAR-4N1 expression cassette. ITR: inverted terminal repeat; hsurP: human survivin promoter. **b.** K562 or **c.** HL60 cells were treated with Ad.sp-E1A or Ad.4N1 at MOIs indicated for 72 h (left chart), or treated with Ad.sp-E1A or Ad.4N1 at 150 MOI for the time period indicated (right chart). Cell viability was analyzed by MTT assay. Values were calculated as percent of PBS control and presented as mean ± SEM. (*p < 0.05) **d.** HL60 cells treated with PBS, Ad.sp-E1A or Ad.4N1 for 48 h were analyzed by Western blot for levels of Bax, Bcl-2, and Caspase 3. GAPDH served as the loading control.

We further engineered a previously reported oncolytic adenovirus Ad.sp-E1A to harbor a cytomegalovirus (CMV) promoter controlled sCAR-4N1 expression cassette, forming a novel oncolytic adenovirus Ad.4N1 (Figure 2A). To evaluate the antiproliferative effect of Ad.4N1, CD47 and survivin-positive leukemia cells K562 [31, 32] and HL60 [33, 34] were treated with Ad.sp-E1A or Ad.4N1. PBS was used as the control. As shown in Figure 2B and 2C, compared to Ad.sp-E1A, Ad.4N1 significantly suppressed the *in vitro* proliferation of both K562 and HL60 cells, at dose- and time-dependent manners. Therefore, data demonstrated that Ad.4N1 successfully infected and induced antiproliferative effect on CD47+ leukemia cells. To further analyze the underlying mechanism of cytotoxicity induced by Ad.4N1, HL60 cells treated with PBS, Ad.sp-E1A, or Ad.4N1 were investigated for apoptotic signaling elements through Western blot. As shown in Figure 2D, Ad.4N1 dramatically induced the upregulation of proapoptotic factor Bax. Interestingly, Ad.4N1 also slightly upregulated the levels of antiapoptotic factor B-cell lymphoma 2 (Bcl-2), but without significant effect on the cleavage of caspase 3. Our data suggest that Ad.4N1 may induce antiproliferative effect on HL60 cells through upregulating Bax, and the upregulation of Bcl-2 may counteract the cytotoxic effect of Ad.4N1.

### Ad.4N1 suppressed leukemia cell proliferation through 4N1-CD47 interaction

**Figure 3:**
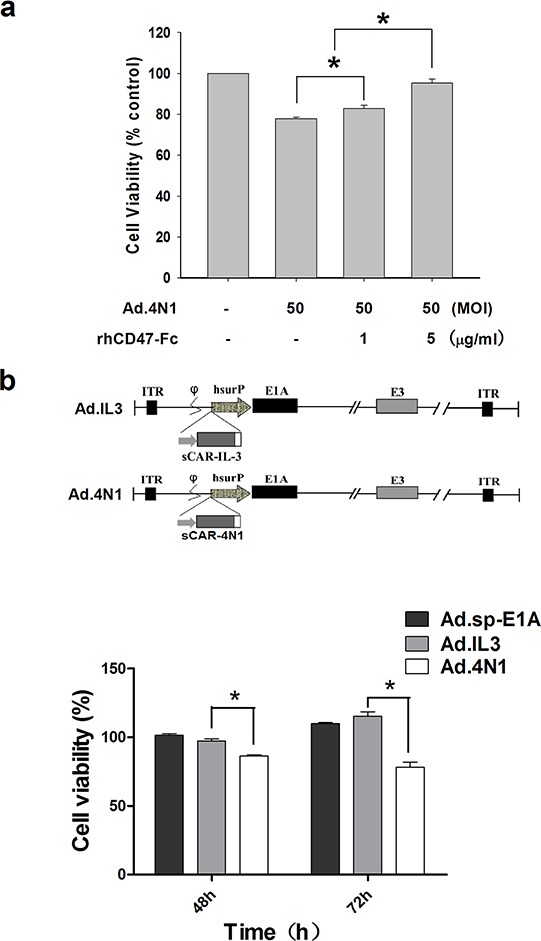
Ad.4N1 suppressed leukemia cell proliferation through the 4N1-CD47 interaction **a.** K562 cells were treated with Ad.4N1 combined with recombinant human CD47 Fc chimera (rhCD47-Fc) at dosages indicated. Cell viability was then determined by MTT assay after 96 h. Values were calculated as percent of PBS control and presented as mean ± SEM. **b.** HL60 cells were treated with Ad.sp-E1A, Ad.IL3, or Ad.4N1 at 200 MOI for the time period indicated. Cell viability was analyzed by MTT assay. Values were calculated as percent of PBS control and presented as mean ± SEM. (*p < 0.05)

To determine that Ad.4N1 infected leukemia cells through CD47, a recombinant human CD47 Fc chimera (rhCD47-Fc) was combined with Ad.4N1 to treat K562 cells, followed by MTT assay for cell viability. As shown in Figure 3A, rhCD47-Fc significantly counteracted with the Ad.4N1 induced proliferation inhibition at a dose-dependant manner, indicating that Ad.4N1 used CD47 as the cell membrane receptor for viral internalization. Furthermore, the antiproliferative effect of Ad.4N1 on HL60 was compared to Ad.IL3, a previously produced oncolytic adenovirus expressing sCAR-IL3 fusion proteins [26]. Results showed that Ad.4N1, but not Ad.IL3, time-dependently suppressed the proliferation of HL60 (Figure 3B). Taken together, our data demonstrated that Ad.4N1 infected and suppressed leukemia cell proliferation through the 4N1-CD47 interaction.

### Ad.4N1 armed with IL-24 elicited higher cytotoxicity to leukemia cells *in vitro* and *in vivo*

**Figure 4:**
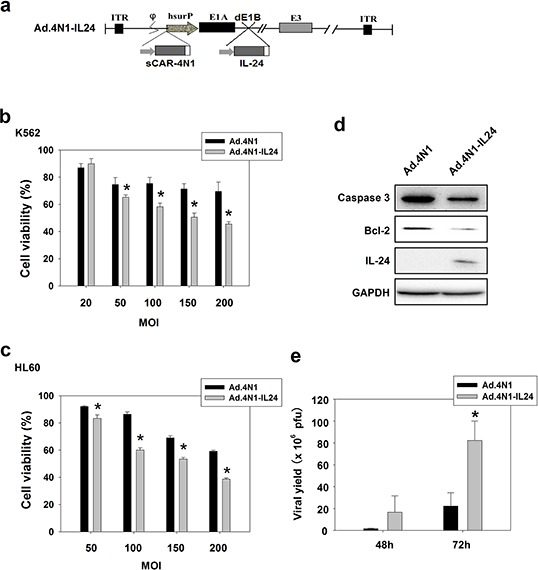
Ad.4N1-IL24 elicited higher cytotoxicity to leukemia cells *in vitro* **a.** Schematic structure of Ad.4N1-IL24. Viral E1B was deleted (dE1B), and the resulted restriction site was used to harbor the gene encoding IL-24. **b.** K562 or **c.** HL60 cells were treated with Ad.4N1 or Ad.4N1-IL24 at MOIs indicated for 72 h. Cell viability was analyzed by MTT assay. Values were calculated as percent of PBS control and presented as mean ± SEM. (*p < 0.05) **d.** HL60 cells treated with Ad.4N1 or Ad.4N1-IL24 at 200 MOI for 48 h were analyzed by Western blot for levels of Caspase 3, Bcl-2, and IL-24. GAPDH served as the loading control. **e.** HL60 cells were treated with Ad.4N1 or Ad.4N1-IL24 at 100 MOI. After 48 h or 72 h, cells were collected and intracellular viruses were released by 3 freeze-thawing cycles. Viral production was determined through TCID50 assay. Values were shown as mean ± SEM. (*p < 0.05)

To further elevate the antiproliferative effect of Ad.4N1, viral E1B was deleted, and a resulted restriction site was used to harbor a CMV promoter controlled human IL-24 expression cassette, forming Ad.4N1-IL24. The schematic structure of the viral genome of Ad.4N1-IL24 was shown in Figure 4A. To evaluate the cytotoxic effect of Ad.4N1-IL24, K562 and HL60 cells were treated with Ad.4N1 or Ad.4N1-IL24 at MOIs indicated, followed by MTT assay. PBS served as the control. As shown in Figure 4B and 4C, Ad.4N1-IL24 suppressed the *in vitro* proliferation of K562 and HL60 at a significantly higher level than Ad.4N1. We then performed further tests on the safety of Ad.4N1-IL24. Because normal human blood cells were unavailable in our studies, lung cancer cell line A549 and normal lung cell line BEASE-2B were analyzed and compared. As shown in Supplementary Figure S1, Ad.4N1-IL24 showed a dramatically lower level of cytotoxicity on BEASE-2B cells than on A549 cells, suggesting the safety of Ad.4N1-IL24. Furthermore, to investigate the underlying mechanism of Ad.4N1-IL24 induced higher cytotoxicity, HL60 cells infected with either Ad.4N1 or Ad.4N1-IL24 were investigated for the levels of caspase 3, Bcl-2, and IL-24. GAPDH served as the loading control. Our data showed that Ad.4N1-IL24 induced not only the expression of IL-24 in HL60 cells, but also more cleavage of caspase 3 as well as a downregulated level of survival factor Bcl-2 (Figure 4D). To determine viral replication capability, HL60 cells were infected with Ad.4N1 or Ad.4N1-IL24 and viral production after the time intervals was examined through TCID50 assay. Data showed that both Ad.4N1 and Ad.4N1-IL24 replicated in HL60 cells. Interestingly, Ad.4N1-IL24 replicated at a dramatically higher rate than Ad.4N1 (Figure 4E). Taken together, our data showed that Ad.4N1-IL24 not only altered apoptotic signaling pathway but also replicated much faster than Ad.4N1, which may be responsible for the higher cytotoxicity of Ad.4N1-IL24.

**Figure 5:**
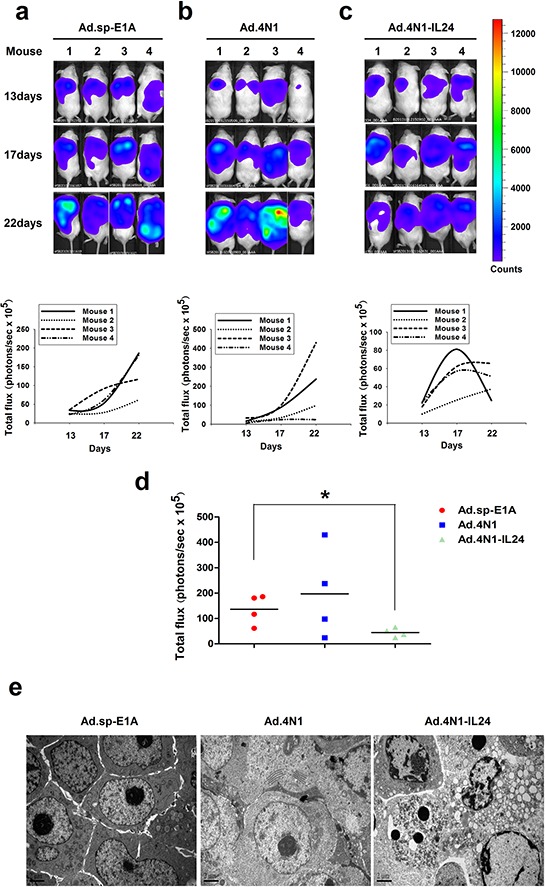
The *in vivo* antileukemia effect of Ad.4N1-IL24 The growth of HL60/Luc cells in mice treated with 2 × 10^9^ pfu of Ad.sp-E1A **a.** Ad.4N1 **b.** or Ad.4N1-IL24 **c.** was evaluated by bioluminescence imaging individually at the day 13, 17, and 22 posttreatment (*n* = 4). The bioluminescence images of HL60/Luc engrafted mice from each group at the day 13, 17, and 22 posttreatment were shown on the top. Quantitative value of leukemia cell burden in individual mouse (photons/sec × 10^5^) was shown at the bottom. **d.** The quantitative analysis of total leukemia cell burden in mice (photons/sec × 10^5^) at the day 22 posttreatment (*p < 0.05). **e.** Apoptotic cells in HL60/Luc xenografts treated with Ad.4N1-IL24 were observed under TEM, as compared to Ad.sp-E1A and Ad.4N1 treatments. Bars show 2 μm.

We then evaluated the *in vivo* antileukemia effect of oncolytic adenoviruses Ad.4N1 and Ad.4N1-IL24. HL60/Luc cells stably expressing fire fly luciferase were engrafted subcutaneously into NOD/SCID mice and monitored for bioluminescence. Mice were then grouped randomly and treated with Ad.4N1, Ad.4N1-IL24, or the control virus Ad.sp-E1A. The growth of HL60/Luc xenograft posttreatment in individual mouse was evaluated by bioluminescence imaging in each group (*n* = 4) (Figure 5A–5C). Values for tumor size were shown in Supplementary Table S1. Data showed that the Ad.4N1-IL24 treatment led to lower cancer cell burden than other treatments, and a significant difference was achieved between Ad.sp-E1A and Ad.4N1-IL24 groups at day 22 (Figure 5D). Apoptotic cells were detected in HL60/Luc xenografts treated with Ad.4N1-IL24 (Figure 5E). Although Ad.4N1-IL24 did not prolong the survival of HL60/Luc xenograft mice as compared to control viruses (data not shown), our data have demonstrated the *in vivo* antileukemia effect of Ad.4N1-IL24 on HL60 leukemia xenografts through reducing cancer cell burden.

## DISCUSSION

Based on our previous studies that oncolytic adenoviruses harboring sCAR-IL3 expression cassette successfully infected CD123+ leukemia cells, we proposed that engineering oncolytic adenoviruses to express sCAR-ligand fusion proteins may provide a universal strategy to redirect oncolytic adenoviruses to various membrane receptors on cancer cells resisting to Ad5 infection. In the work presented, we demonstrated that Ad.4N1 successfully infected and suppressed *in vitro* proliferation of CD47+ K562 and HL60 leukemia cells. The *in vitro* infection and antiproliferative effect of Ad.4N1 was determined to be mediated by the 4N1-CD47 interaction. We further engineered Ad.4N1 to harbor a gene encoding IL-24, forming Ad.4N1-IL24, which successfully elicited *in vivo* antileukemia effect on a HL60 xenograft model. Although Ad.4N1 did not suppress *in vivo* leukemia growth as shown in Figure 5, our data have demonstrated that it could act as a useful tool for delivering anticancer genes into CD47+ leukemia cells. Therefore, our data indicated that oncolytic adenoviruses carrying gene encoding sCAR-4N1 fusion protein is a novel strategy to redirect oncolytic adenoviruses to CD47+ cancer cells.

TSP-1 has been extensively studied as an antitumor factor due to its antiangiogenic effect on papillary thyroid carcinoma [35], breast cancer [36], and melanoma [37]. In mammary tumor, TSP-1 was shown to inhibit angiogenesis, but promote tumor metastasis [38]. TSP-1 secreted by glioma cells was also linked to promoting cell motility, suggesting an effect of TSP-1 in enhancing glioma metastasis [39]. CD36 has been recognized as the primary receptor for TSP-1 in inhibiting angiogenesis [40] as well as promoting metastasis [38], and the type I repeat domain of TSP-1 mediates the TSP-1-CD36 interaction. In addition to CD36, TSP-1 also acts as a ligand for CD47, and the C-terminal domain 4N1 peptide of TSP-1, is responsible for the TSP-1-CD47 binding [28, 41]. The 4N1 peptide has been shown to induce apoptosis or autophagy in various cancer cells, including chronic lymphocytic leukemia cells [42], breast cancer cells [43], and some Ras-expressing cancer cells [44]. However, 4N1 was also shown to inhibit apoptosis induced by camptothecin or doxorubicin in thyroid carcinoma cells [41]. In our results, sCAR-4N1 expressing oncolytic adenovirus Ad.4N1 elicited cytotoxicity to K562 and HL60 leukemia cells *in vitro*, but did not suppress the *in vivo* growth of HL60 cells. Our results and others have suggested diverse effects of the 4N1-CD47 interaction on different cancer cells under varied conditions. A previous study showed that the 4N1-CD47 interaction led to the binding of CD47 with αIIbβ3 integrin, which subsequently changed αIIbβ3 integrin into a high affinity state and promoted cell adhesion [45]. In fact, different from *in vitro* cultured leukemia cells, *in vivo* leukemia cells adhere to various extracellular matrix components and other cell types. Therefore, we propose here that cell adhesion status modulated by the 4N1-CD47 interaction could in turn become an important factor inhibiting the oncolytic adenoviruses as well as other therapeutic agents induced apoptosis signaling, pending further investigations.

Cytokine mda-7/IL-24 has been extensively investigated for selective antitumor effect on prostate cancer [46], lung cancer [47], ovarian cancer [48], breast carcinoma [49], colorectal cancer [50], and glioma [51]. Through gene delivery by a replication-deficient adenovirus vector (Ad.mda-7/IL-24), the antitumor effect of IL-24 is achieved through both ectopic expression [47] and secretion [52]. IL-24 induces cancer selective apoptosis through interacting with endoplasmic reticulum (ER) chaperone protein BiP/GRP78 [53], activating Fas/FasL signaling pathway [48], as well as reduction in myeloid cell leukemia-1 (Mcl-1) expression [54]. Ad.mda-7/IL-24 has entered clinical studies, and significant effectiveness and safety have been demonstrated [55]. However, some studies suggested that conditionally replicative oncolytic adenoviruses armed with IL-24 would be more effective [56, 57]. Although high level IL-24 was linked to promoting chronic lymphocytic leukemia cell survival through activating p38 MAPK [58], several other preclinical studies demonstrated that the gene delivery of IL-24 through oncolytic adenoviruses elicited significant antileukemia effect *in vitro* and *in vivo* [19, 59]. In our *in vitro* and *in vivo* studies, the gene delivery of IL-24 through a CD47 targeting oncolytic adenovirus significantly enhanced antileukemia effect than the vector alone, which has not been shown in other studies. Furthermore, we demonstrated that the level of survival factor Bcl-2 upregulated by Ad.4N1 could be suppressed by the deletion of viral E1B and further harboring IL-24, and Ad.4N1-IL24 replicated at a dramatically higher rate than Ad.4N1, which may be responsible for the higher antiproliferative effect of Ad.4N1-IL24 on leukemia cells. Therefore, our results demonstrated that CD47-retargeted oncolytic adenoviruses armed with IL-24 could be valuable for the treatment of CD47+ leukemia.

In general, we provided a novel strategy to redirect oncolytic adenoviruses to CD47+ leukemia cells though carrying a sCAR-4N1 expression cassette in the viral genome, forming Ad.4N1. The *in vitro* infection and cytotoxicty of Ad.4N1 in leukemia cells was determined to be mediated by the 4N1-CD47 interaction. Furthermore, Ad.4N1 armed with the gene encoding IL-24 achieved significant antileukemia effects both *in vitro* and *in vivo*. Our data suggest that further clinical investigation into the antileukemia effect of Ad.4N1-IL24 may provide a novel antileukemia agent for future CD47+ leukemia therapies.

## MATERIALS AND METHODS

### Cells

Leukemia cell lines K562 and HL60 were obtained from American Type Culture Collection (ATCC, Rockville, MD, USA). Cells were routinely cultured in RPMI1640 (Hyclone Laboratories, Logan, UT, USA) supplemented with 10% fetal bovine serum (FBS, Hyclone Laboratories). The human embryonic kidney cell HEK293 was obtained from Microbix Biosystems (Toronto, ON, Canada) and cultured in Dulbecco's modified Eagle's medium (DMEM; GIBCOBRL, Grand Island, NY, USA) supplemented with 10% fetal bovine serum. All cells were kept at 37°C in a 95% air 5% CO_2_ humidified incubator.

### Recombinant protein preparation

To construct plasmid pOE30-sCAR-4N1, a two-step PCR was used to generate *sCAR-4N1* gene from a previously reported plasmid pQE30-sCAR-IL3 [26]. The first PCR used pOE30-sCAR-IL3 as the template. The primers were 5′-CCCAAGCTTATGGCGCTCCTGCTGT GCTT-3′ and 5′-TCACAACATAAAAGCGGGATCCAG GGGCG-3′. The second PCR used the product of the first PCR as the template. The primers were 5′-CCCAAGCTTATGGCGCTCCTGCTGTGCTT-3′ and 5′-CCGAAGCTT TTAGGATCCAGGGGCG GAAG-3′. The product of the second PCR was inserted into the *Hind*III site of pQE30 to generate pOE30-sCAR-4N1. The his-tagged sCAR-4N1 fusion proteins were produced in *Escherichia coli* M15, extracted from inclusion bodies, and refolded by standard methods.

### Construction of recombinant adenoviruses

The plasmid pAd.sp-E1A was constructed previously [27]. The sCAR-4N1 expression cassette was inserted into pAd.sp-E1A to generate pAd.sp-E1A-sCAR-4N1. The *Nhe*I-*Ale*I fragments from pAd.sp-E1A-sCAR-4N1 were replaced into the *Nhe*I-*Ale*I regions in plasmids pAd-△E1B-IL24, to generate pAd.sp-E1A-sCAR-4N1-△E1B-IL24. Plasmids pAd.sp-E1A-sCAR-4N1 or pAd.sp-E1A-sCAR-4N1-△E1B-IL24 was then co-transfected with an adenovirus packing plasmid pBHGE3 (Microbix Biosystems, Toronto, Canada) into HEK293 cells to generate oncolytic adenoviruses Ad.4N1 and Ad.4N1-IL24 through homologous recombination. The recombinant adenoviruses were isolated through three rounds of plaque purification in HEK293 cells. Large-scale purification of adenoviruses was performed by ultracentrifugation with cesium chloride. The viral titers were determined by TCID50 (median tissue culture infective dose) assay in HEK293 cells.

### Cytotoxicity assay

Leukemia cells were plated on 96-well plates at 2 × 10^4^ per well. Cells in 6–7 replication wells were then infected with viruses at indicated MOIs. PBS was used as a control. The cell viability was determined by 3-(4,5-dimethylthiazol-2-yl)-2,5-diphenyltetrazolium bromide (MTT) assay.

For blocking assays, K562 cells were plated on 96-well plates at 2 × 10^4^ per well. Cells were then treated with viruses at the indicated MOIs in combination with recombinant human CD47 Fc chimera (R&D systems, Minneapolis, MN, USA) at concentrations indicated. Cell viability was then determined by MTT assay after 96 h.

### Western blotting analysis

The cell extracts were subjected to SDS-PAGE and electroblotted onto nitrocellulose membranes. The membranes were then blocked with Tris-buffered saline and Tween 20 contaning 5% of bovine serum albumin at room temperature for 1 h, followed by incubation with primary antibodies overnight at 4°C. The membranes were then washed and incubated with secondary antibodies for 1 h at room temperature. After washing with Tris-buffered saline, the bands were detected under a Tanon 5500 chemiluminescence image system (Tanon Inc., Shanghai, China).

Rabbit anti-caspase 3, Bcl-2, Bax, and GAPDH antibodies were purchased from Cell Signaling Technology Inc. (Danvers, MA, USA). Goat antibody against IL-24 was purchased from R&D systems Inc. (Minneapolis, MN, USA). The HRP conjugated goat anti-rabbit and donkey anti-goat antibodies were purchased from MultiSciences (Lianke) Biotech Co., Ltd. (Hangzhou, China).

### Animal experiments

Male NOD/SCID mice at 4–5 weeks of age were used for leukemia xenograft. HL-60/Luc cells at 6 × 10^6^ cells/mouse were injected subcutaneously into the mice on the back. When leukemia burdens reached about 1 × 10^5^ photons/sec, mice were randomly grouped and in situ injected with 2 × 10^9^ plaque-forming units (pfu) of oncolytic adenoviruses. Mice were injected with D-Luciferin, and bioluminescence was recorded under a Caliber IVIS kinetics (Caliper life sciences, USA). Regions of interest were assigned through the IVIS software and reported as area flux (photons/sec), defined by radiance (photons/sec/cm^2^/steradian).

### Ethnic statement

All animal studies were approved by the Institutional Animal Care and Use Committee (IACUC) of Zhejiang Chinese Medical University, Zhejiang, China.

### Statistical analysis

Differences among the treatment groups were assessed by student's *t*-test. *P* < 0.05 was considered significant.

## SUPPLEMENTARY FIGURE AND TABLE


