# Supplementary material for: CD47-retargeted oncolytic adenovirus armed with melanoma differentiation-associated gene-7/interleukin-24 suppresses in vivo leukemia cell growth
Source: Oncotarget. 2015 Nov 2;6(41):43496–507. doi: 10.18632/oncotarget.6292 (PMC4791246; doi:10.18632/oncotarget.6292)
Supplement: Supplementary file 1 [file oncotarget-06-43496-s001.pdf]

SUPPLEMENTARY FIGURE AND TABLE

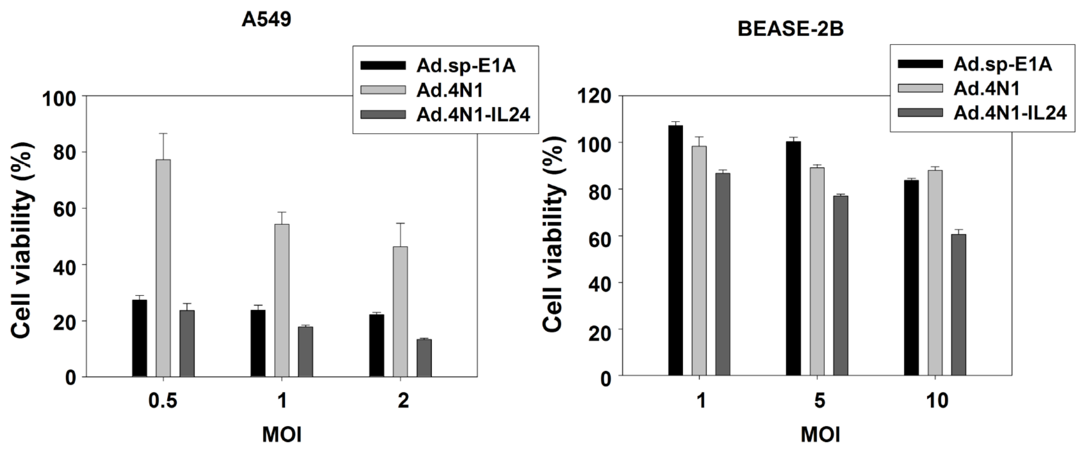

Supplementary Figure S1: The cytotoxic effect of Ad.4N1 and Ad.4N1-IL24 on A549 and BEASE-2B cells. A549 and BEASE-2B cells were treated with oncolytic adenovirus as indicated for 96 h followed by MTT assay. Values were calculated as percent of PBS control and presented as mean ± SEM.

Supplementary Table S1: Values for tumor size measured by bioluminescence imaging (× 10^5)

Viruses	13 days	17 days	22 days	Mouse No.
Ad.sp-E1A	33.48	53.01	186.20	Mouse 1
	24.74	27.80	61.35	Mouse 2
	34.77	91.65	116.70	Mouse 3
	22.72	61.47	180.80	Mouse 4
Ad.4N1	16.48	89.73	237.70	Mouse 1
	14.92	35.01	98.08	Mouse 2
	32.99	100.30	429.80	Mouse 3
	5.875	24.17	24.17	Mouse 4
Ad.4N1-IL24	22.08	81.20	24.80	Mouse 1
	9.946	25.30	37.46	Mouse 2
	17.94	62.24	65.53	Mouse 3
	23.01	57.15	51.32	Mouse 4
